# Safety and Efficacy Studies of Vertebroplasty, Kyphoplasty, and Mesh-Container-Plasty for the Treatment of Vertebral Compression Fractures: Preliminary Report

**DOI:** 10.1371/journal.pone.0151492

**Published:** 2016-03-10

**Authors:** Chen Chen, Donghua Li, Zhiguo Wang, Tong Li, Xunwei Liu, Jian Zhong

**Affiliations:** 1 Department of Orthopedic Surgery, The General Hospital of Jinan Command, Jinan, Shandong Province, 250031, People’s Republic of China; 2 Radiology Department of Yuncheng County People's Hospital, Yuncheng, Shandong Province, 274700, People’s Republic of China; 3 Department of Medical Image, The General Hospital of Jinan Command, Jinan, Shandong Province, 250031, People’s Republic of China; 4 College of Food Science & Technology, Shanghai Ocean University, Shanghai, 201306, People's Republic of China; University of Zaragoza, SPAIN

## Abstract

To evaluate the clinical safety and efficacies of percutaneous vertebroplasty (PVP), percutaneous kyphoplasty (PKP), and percutaneous mesh-container-plasty (PMCP) for the treatment of vertebral compression fractures (VCFs), a retrospective study of 90 patients with VCFs who had been treated by PVP (n = 30), PKP (n = 30), and PMCP (n = 30) was conducted. The clinical efficacies of these three treatments were evaluated by comparing their PMMA cement leakages, cement patterns, height restoration percentages, wedge angles, visual analogue scales (VAS), and oswestry disability index (ODI) at the pre- and post-operative time points. 6.67%, 3.33%, and 0% of patients had PMMA leakage in PVP, PKP, and PMCP groups, respectively. Three (solid, trabecular, and mixed patterns), two (solid and mixed patterns), and one (mixed patterns) types of cement patterns were observed in PVP, PKP, and PMCP groups, respectively. PKP and PMCP treatments had better height restoration ability than PVP treatment. PVP, PKP, and PMCP treatments had significant and similar ability in pain relief and functional recovery ability for the treatment of VCFs. These results indicate minimally invasive techniques were effective methods for the treatment of VCFs. Moreover, these initial outcomes suggest PMCP treatment may be better than both PVP treatment and PKP treatment.

## Introduction

Vertebral compression fractures (VCFs) are seen increasingly in clinics in the world. VCFs cause chronic pain, sleep loss, depression, and significant limitation in quotidian activities.[1] Moreover, they are commonly associated with an increased risk of further painful VCF, resulting in height loss, kyphosis, and increased risk of nonvertebral fractures [2]. Currently, there are three kinds of treatments for VCFs are used in clinics:[3–6] traditional conservative treatment, traditional surgical treatment, minimally invasive treatment. Compared to the other two treatments, minimally invasive treatments have been increasingly used and will be the mainstream methods for the treatment of VCFs in the future due to its smaller incision, shorter treatment time, less blood loss, less pain, shorter recovery time, and shorter hospitalization [7–9].

During the past thirty years, two kinds of minimally invasive techniques (PVP and PKP) were mainly introduced for VCF treatments:[10] percutaneous vertebroplasty (PVP) and percutaneous kyphoplasty (PKP). PVP was first described by Gilibert et al. in 1987 as a treatment for vertebral haemangiomas [11] and then was pioneered by the same group as a treatment of osteoporotic VCF [7]. During the PVP process, the Polymethyl methacrylate (PMMA) cement is directly injected into the fractured vertebral body. PMMA cement is dispersed and cured in the vertebral body, and therefore the fractured vertebral body is strengthened and stabilized. As a modified form of PVP, PKP is similar to PVP except that commercial inflatable bone tamp or Sky-bone expander is percutaneously inserted into the fractured vertebral body, and then is inflated, deflated, and withdrawn to create a cavity and to restore the fractured vertebral body height prior to bone cement injection.[8, 9] PVP and PKP have been widely applied for the treatment of VCFs. However, the benefits and shortcomings of these two techniques are still debated such as height restoration and bone cement leakage [12–14].

Percutaneous mesh-container-plasty (PMCP) is an emerging minimally invasive technique and is developed by referring to PVP and PKP. In PMCP technique, a cavity is formed in the fractured vertebral body by applying a bone expansion brace to cut the bone tissues. After withdrawn of the bone expansion brace, mesh container is advanced into the cavity and PMMA cement is injected into the mesh container. During the cement injection process, the mesh container is expanded and reaches the edge of the cavity. The continuous cement injection makes the mesh container produce a pressure to the surrounding bone tissues and the height of the fractured vertebral was gradually restored. When the perfusion pressure reaches a certain degree, bone cement leaks outside of the mesh container from the meshes and enters the bone trabeculae, and therefore the bone trabeculae are strengthened and stabilized.

It is necessary to evaluate the clinical safety and efficacy of PVP, PKP, and PMCP for the treatment of VCFs, which will be helpful for the spinal surgeon community to know the PMCP treatment and to further explore the clinical use of PVP, PKP, and PMCP for the treatment of VCFs. The purpose of this retrospective study was to systematically evaluate the clinical safety and efficacy of PVP, PKP, and PMCP for the treatment of VCFs.

## Results

### Patients information

90 patients (PVP:PKP:PMCP = 1:1:1) with single VCF were treated with PVP, PKP, and PMCP in a randomized double-blind way. The clinical characteristics of the 90 patients with single VCF were summarized in Table 1. Among the PVP, PKP, and PMCP groups, there were no statistical significances in sex, age, bone mineral density T-score, and distribution of fractured vertebral bodies.

**Table 1:** Clinical characteristics of the 90 patients with single VCF in this study.

Group	Number of patients (vertebral bodies)	Mal/Female	Age	Bone mineral density T-score	Distribution of fractured vertebral bodies						
					T_10_	T_11_	T_12_	L_1_	L_2_	L_3_	L_4_
PVP group	30	5/25	69.78±8.71	-2.86±0.79	1	2	9	8	4	4	2
PKP group	30	7/23	70.23±6.54	-2.91±0.64	1	3	8	6	5	5	2
PMCP group	30	4/26	70.52±7.13	-2.87±0.67	2	2	8	8	5	4	1
*t* value or χ^2^ value	—	χ^2^ = 0.122	*t* = 0.678	*t* = 0.367	—						
*P* value	—	0.941	0.512	0.751	0.990						

Note----There were no significant differences (*P*>0.05) among these three groups.

### Surgical results

**Fig 1:**
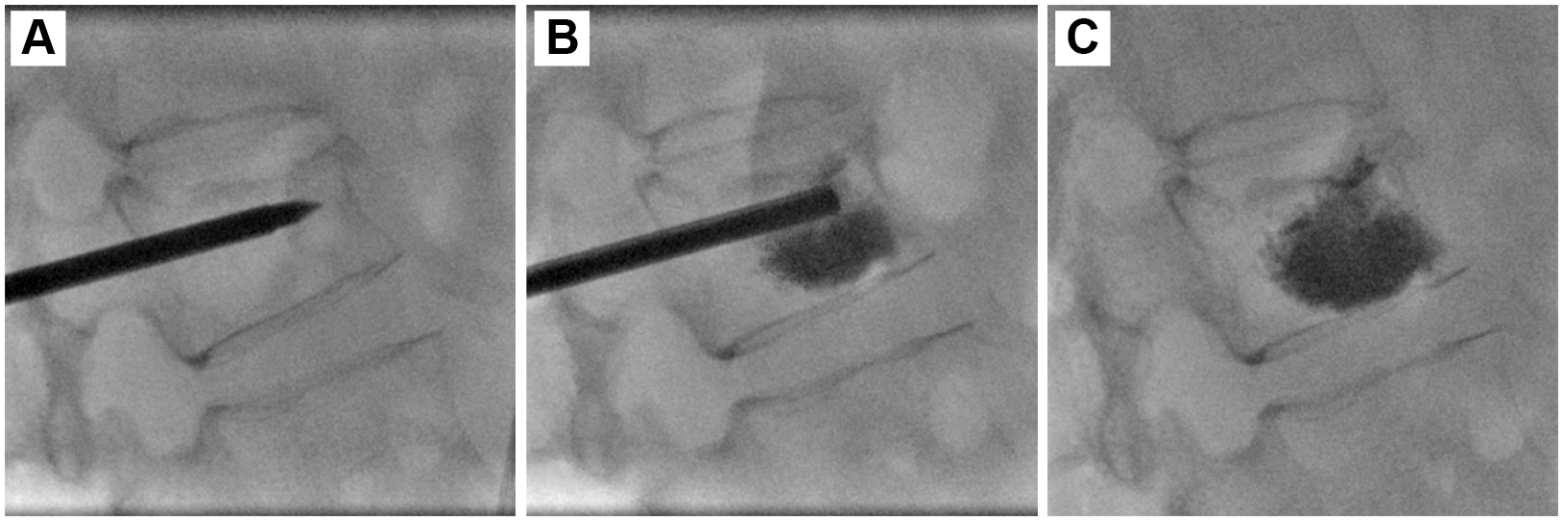
PVP surgical procedure for the treatment of a 61-year-old male patient with single VCF in L1 vertebra. (A): A puncture needle entered into the anterior column of the wedge L1 vertebra via the left pedicle. (B): PMMA bone cement was injected into the fractured vertebral body via the puncture needle. PMMA cement dispersed into the fractured vertebral body and the vertebral height was uplifted. (C): After the PMMA injection, the puncture needle was withdrawn.

**Fig 2:**
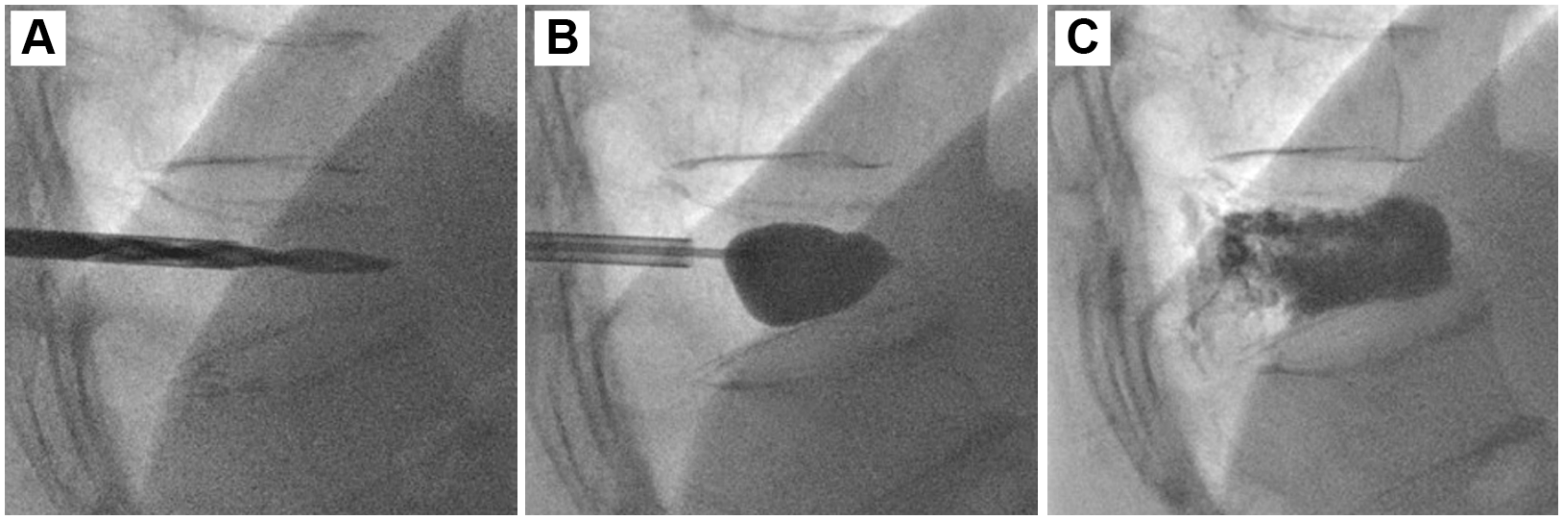
PKP surgical procedure for the treatment of a 73-year-old female patient with single VCF in T11 vertebra. (A): A puncture needle entered into the anterior column of the wedge T11 vertebra via the left pedicle and a bone drill was placed in to drill a circular hole in the fractured vertebral body as a working channel. (B): After withdrawing the bone drill, an inflatable bone tamp was placed into the working channel and was slowly inflated, which induced the uplift of the vertebra height. (C): After PMMA cement injection, the puncture needle was withdrawn.

**Fig 3:**
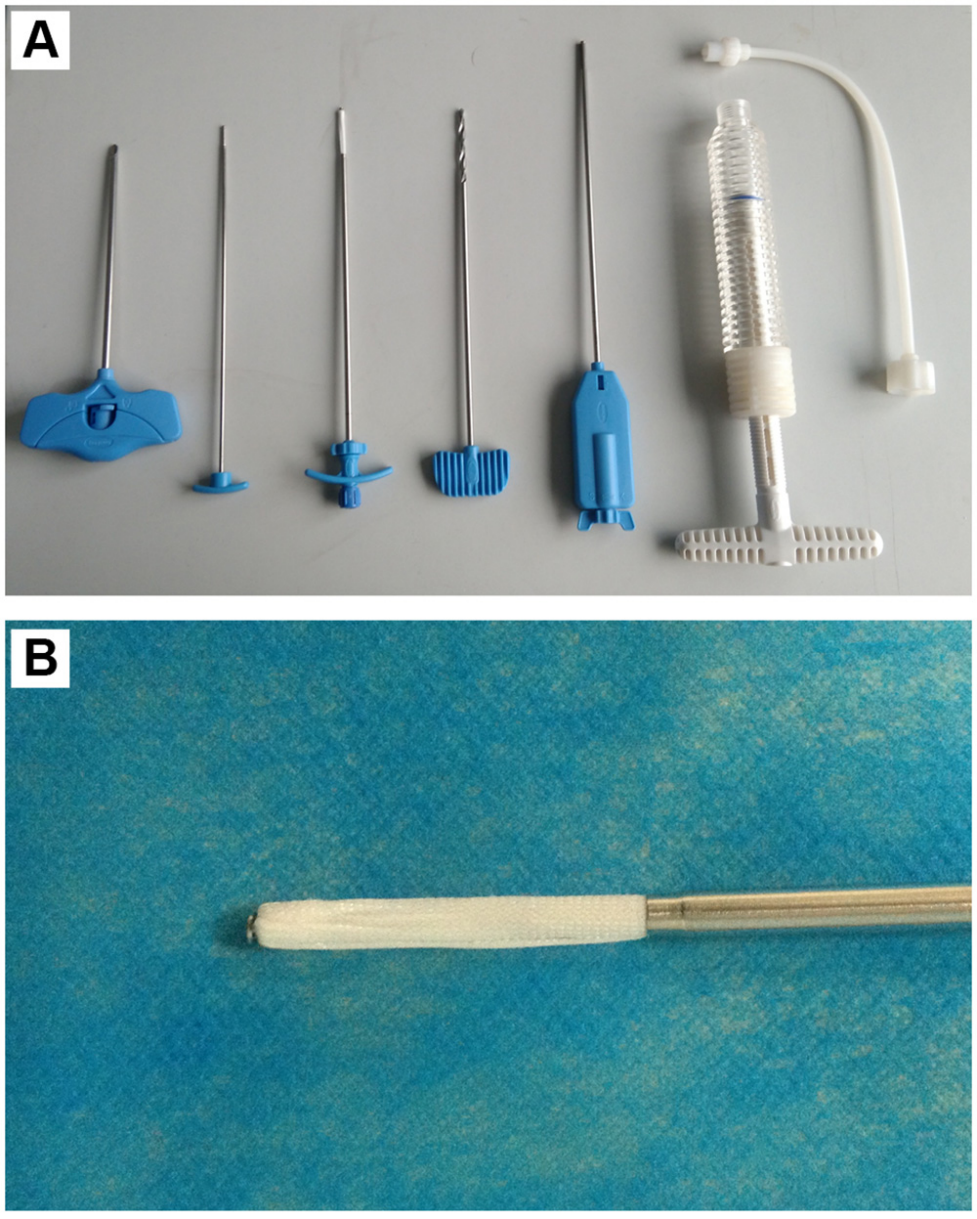
PMCP instruments. (A): puncture needle, push rod for mesh container, mesh container and its delivery instrument, bone drill, expansion brace, cement pump, and connection tube (from left to right). (B) the enlarged mesh container.

**Fig 4:**
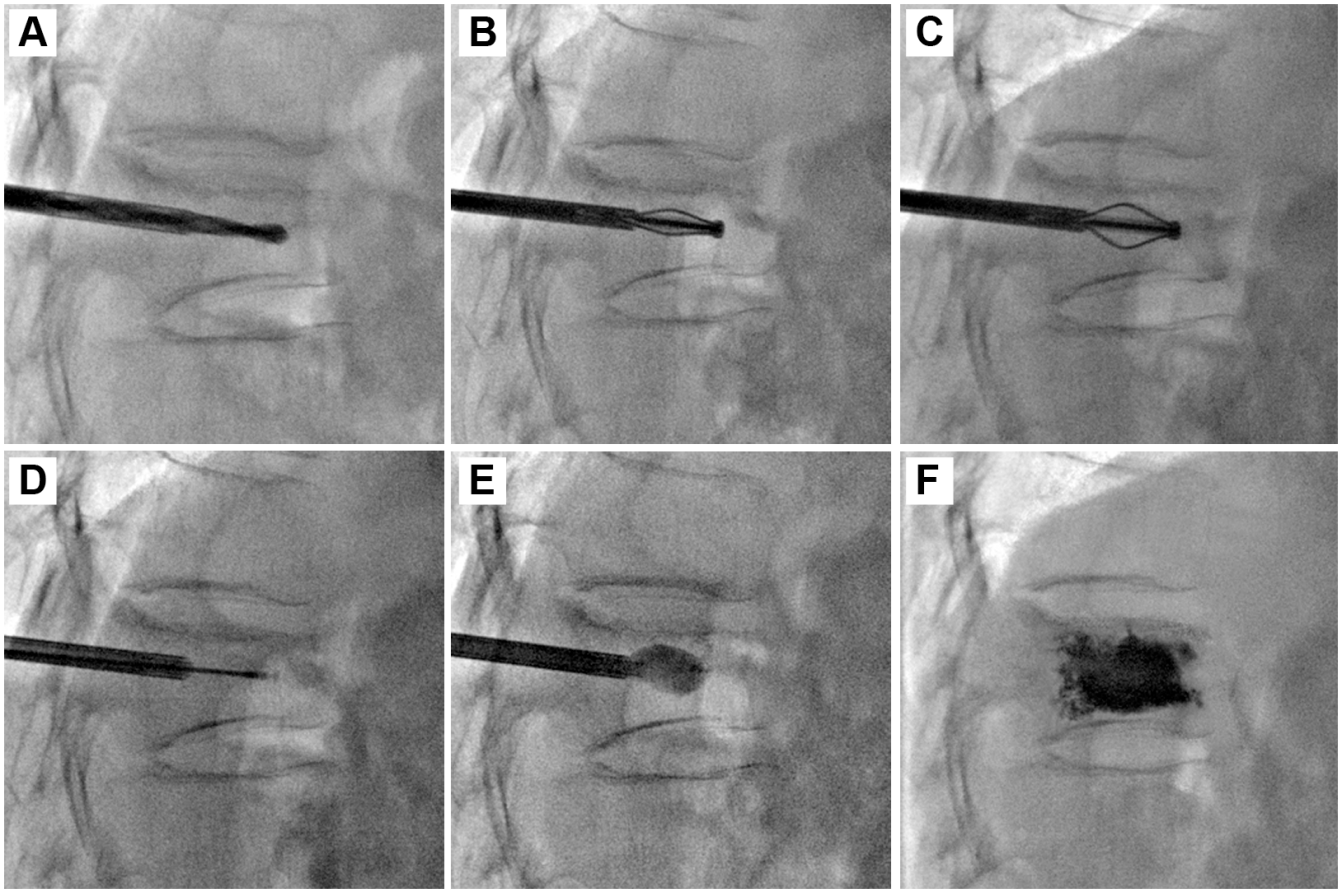
PMCP surgical procedure for the treatment of a 68-year-old female patient with single VCF in T12 vertebra. (A): A puncture needle entered into the anterior column of the wedge T12 vertebra via the left pedicle and a bone drill was placed in to drill a circular hole in the fractured vertebral body as a working channel. (B): An expansion brace was placed into the working channel. The surrounding bone tissues were cut by expanding the spring leaves and rotating the bone expansion brace. (C): After withdrawing the expansion brace, a mesh container was placed into the cavity (indicated by the black arrow). (D): PMMA cement was injected into the mesh container and the mesh container was slowly inflated. (E): The mesh container was continued to be inflated by continuous PMMA injection. (F) PMMA cement leaked outside of the mesh container from the meshes. After PMMA cement injection, the puncture needle was withdrawn.

**Fig 5:**
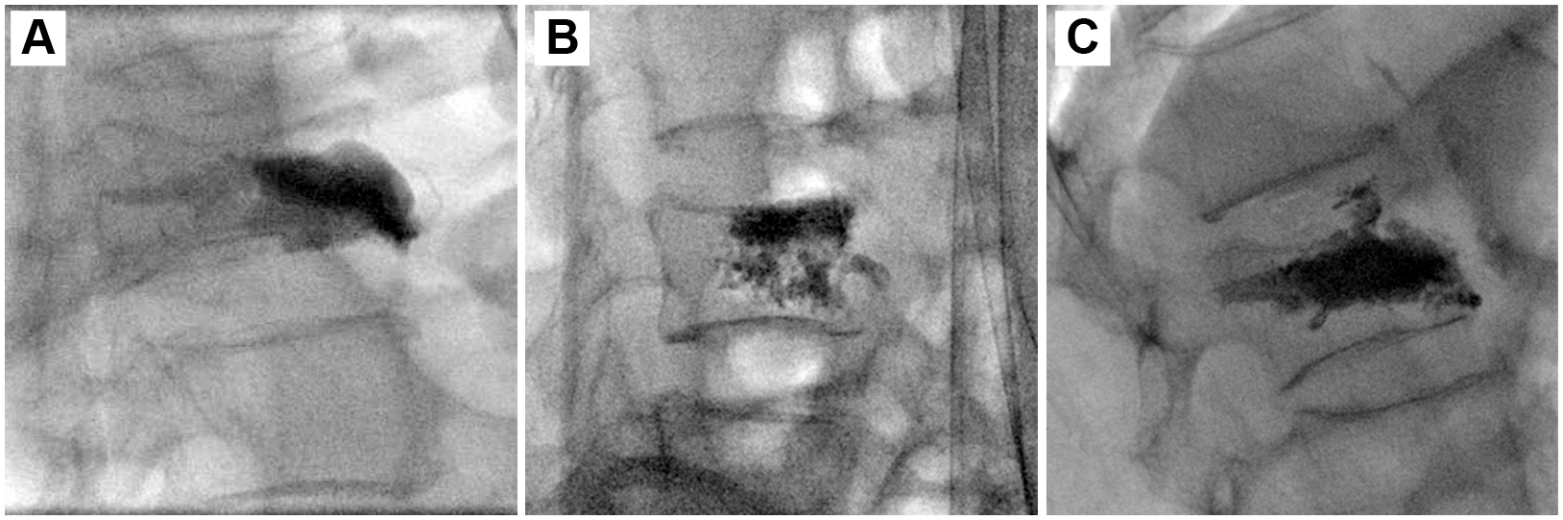
Radiographs of PMMA cement leakage. (A) In a case of PVP treatment, PMMA leaked into vein in a linear way. (B) In a case of PVP treatment, PMMA leaked outside of the treated vertebra. (C) In a case of PKP treatment, PMMA leaked from the broken endplate to the intervertebral disc.

The fractured vertebral bodies of the 90 patients were punctured through a unilateral transpedicular approach to perform PVP (Fig 1), PKP (Fig 2), or PMCP (Figs 3 and 4). The puncture success rate was 100%. The amount of injected PMMA cement per vertebra ranged from 2.5 to 4.0 mL. After surgeries, CT was performed immediately to assess PMMA cement leakage. 6.67% (2/30), 3.33% (1/30), and 0% (0/30) of patients had PMMA leakage in PVP, PKP, and PMCP groups (Table 2), respectively. The Chi-sqaured test showed no obvious difference (p = 0.355, χ^2^ = 2.069). Further large sample numbers should be performed for statistical analysis. In the two leakage cases of PVP group, PMMA leaked into vein in a linear way (Fig 5A) or outside of the treated vertebra (Fig 5B). In the one leakage case of PKP group, PMMA leaked from the broken endplate to the intervertebral disc (Fig 5C). There were no clinical symptoms occurred in the three patients with PMMA leakage. According to above analyses, PMCP treatment had better safety than PVP and PKP treatments.

**Table 2:** Surgical results of PVP, PKP, and PMCP for the treatment of the 90 patients with VCFs in this study.

Group	Number (percentage) of bone cement leakage	Type and number (percentage) of patterns of cement		
		Solid patterns	Trabecular patterns	Mixed patterns
PVP group	2 (6.67%)	7 (23.3%)	6 (20%)	17 (56.7%)
PKP group	1 (3.33%)	23 (76.7%)	0 (0%)	7 (23.3%)
PMCP group	0	0 (0%)	0 (0%)	30 (100%)

The cement patterns after the surgeries were summarized in Table 2. PVP group had 23.3% (7/30) solid patterns, 20% (6/30) trabecular patterns, and 56.7% (17/30) mixed patterns. PKP group had 76.7% (23/30) solid patterns and 23.3% (7/30) mixed patterns. PMCP group had 100% (30/30) mixed patterns.

### Height restoration and wedge angle

The fluoroscopic spot radiographs were reviewed to measure height restoration percentages and wedge angles before and after the surgeries (Table 3). There were significant differences (P<0.05) in height restoration percentage between anterior and central vertebral body after PVP, PKP, or PMCP treatments. Moreover, there was significant difference (P<0.05) in wedge angles before and after PVP, PKP, or PMCP treatment. Moreover, long-term follow-up results showed the wedge angle did not obviously change even after 6 months. After 6 months, no damage was observed in the two vertebrae that were adjacent to the treated vertebrae for the three treatments. Therefore, all PVP, PKP, and PMCP treatments could significantly restore the height of the fractured vertebral body. PKP group and PMCP group had no significant differences (P>0.05) in height restoration percentages and wedge angles. However, these two groups had significantly (P<0.05) higher height restoration percentages and wedge angles than PVP group, which indicated that PKP and PMCP treatments had better height restoration ability of the treated vertebral body than PVP treatment. Therefore, though PVP, PKP, and PMCP treatments had obvious height restoration ability, PKP and PMCP treatments had better height restoration ability.

**Table 3:** Height restoration comparisons of PVP, PKP, and PMCP for the treatment of the 90 patients with VCFs in this study.

Group	Percentage of height restoration of fractured vertebral bodies after the surgeries*		Wedge angle*				
	Anterior vertebral body (%)	Central vertebral body (%)	Pre-procedure (°)	Post-procedure (°)	1 m (°)	3 m (°)	6 m (°)
PVP group	3.56±2.84	5.37±4.51	20.57±10.37	16.03±9.28	16.11±8.97	16.26±9.21	16.43±9.33
PKP group	12.17±11.54	22.58±15.72	21.19±10.78	9.73±5.92	9.78±6.12	9.80±5.67	9.91±6.32
PMCP group	12.67±10.69	21.02±14.83	20.53±9.91	11.27±7.36	11.31±7.48	11.34±7.35	11.61±7.46

* A paired *t* test was used for the statistical analysis. There were significant differences (*P*<0.05) between pre-procedure and post-procedure of these three groups. There were significant differences (*P*<0.05) between PVP group and other two groups. There were no significant difference (*P*>0.05) between PKP group and PMCP group.

### Pain and function evaluation

As shown in Table 4, pain relief and functional recovery of the 90 patients were evaluated by VAS and ODI, respectively. There were significant differences (P<0.05) in VAS scores and ODI scores before and after treatments. Moreover, long-term follow-up results showed VAS scores and ODI scores did not obviously change even after 6 months. Therefore, all PVP, PKP and PMCP treatments had significant pain relief and functional recovery ability. There were no significant differences (P>0.05) in VAS scores and ODI scores among PVP, PKP, and PMCP treatment, which indicated that these three techniques had no obvious difference in pain relief and functional recovery ability. Therefore, PVP, PKP, and PMCP treatments had significant and similar ability in pain relief and functional recovery ability for the treatment of VCFs.

**Table 4:** Pain and function evaluation comparisons of PVP, PKP, and PMCPfor the treatment of the 90 patients with VCFs in this study.

Group	VAS*					ODI*				
	Pre-procedure	3 d	1 m	3 m	6 m	Pre-procedure	3 d	1 m	3 m	6 m
PVP group	8.31±1.25	1.80±1.03	1.86±0.78	2.06±0.82	2.21±1.23	71.92±4.89	23.42±2.69	24.40±3.74	24.13±4.19	24.93±3.96
PKP group	8.29±1.12	1.81±1.13	1.86±1.54	2.23±1.29	2.40±1.52	73.15±4.38	24.42±4.08	24.56±3.54	24.36±3.98	27.67±12.80
PMCP group	8.32±1.37	1.77±1.36	1.83±1.46	2.02±1.23	2.18±1.37	74.23±4.87	23.94±2.71	23.86±3.72	24.09±3.67	24.20±3.54

* A paired *t* test was used for the statistical analysis. There were significant differences (*P*<0.05) between pre-procedure and post-procedure of these three groups. There were no significant differences (*P*>0.05) among these three groups.

### Long-term DSA follow-up

**Fig 6:**
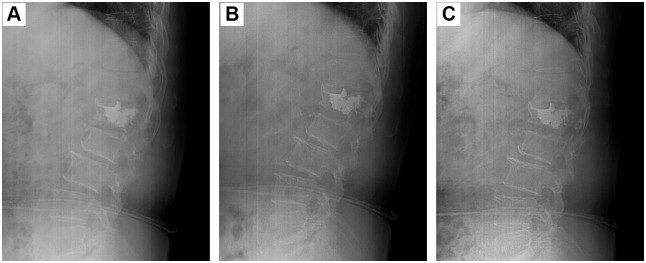
Long-term observation of PVP surgery for the treatment of a 74-year-old female patient with single VCF in L1 vertebra. (A): 1 month; (B): 3 months; (C): 6 months.

**Fig 7:**
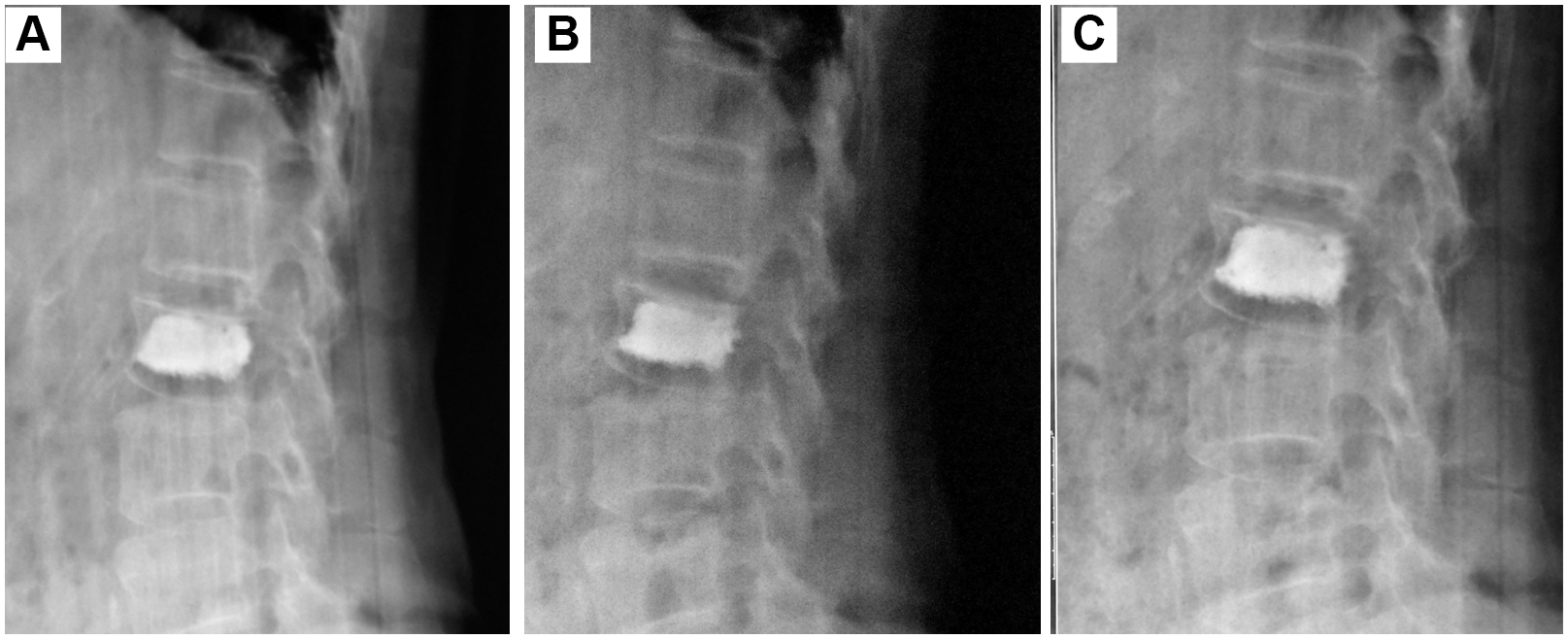
Long-term observation of PVP surgery for the treatment of a 70-year-old female patient with single VCF in L2 vertebra. (A): 1 month; (B): 3 months; (C): 6 months.

**Fig 8:**
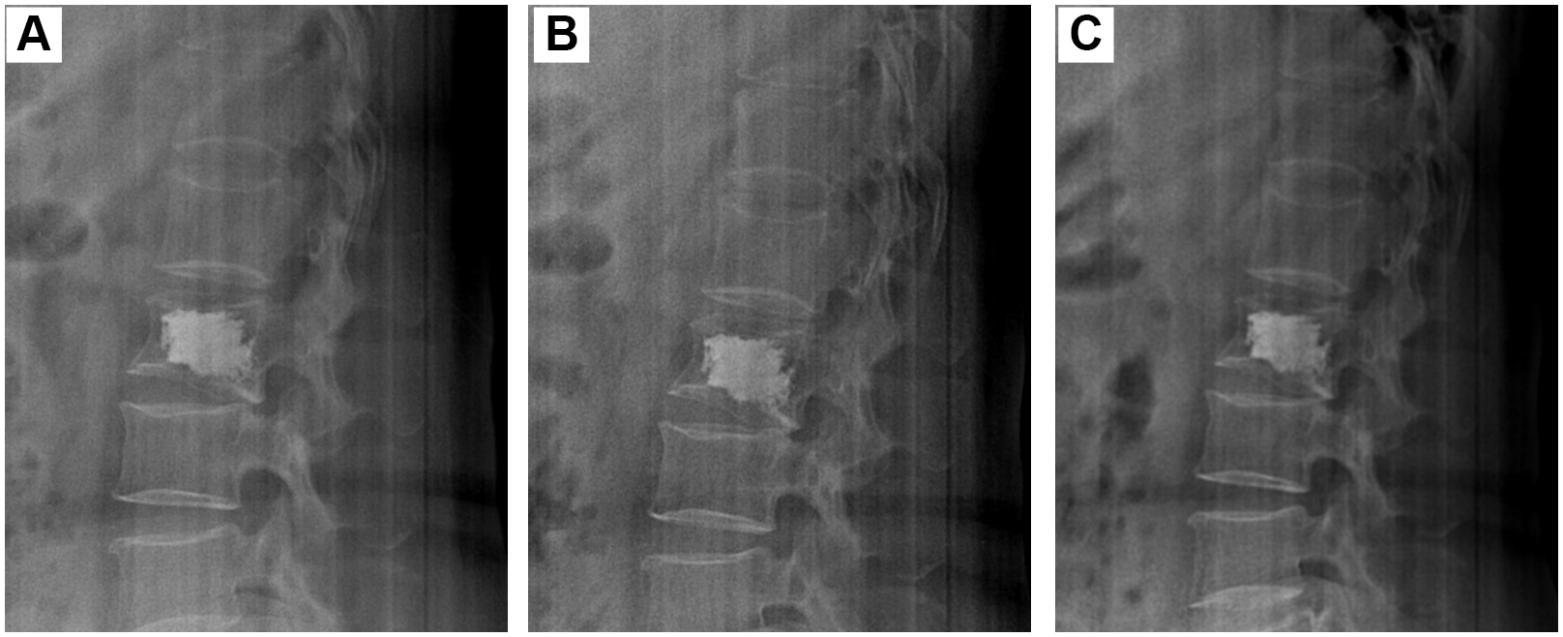
Long-term observation of PVP surgery for the treatment of a 67-year-old male patient with single VCF in L2 vertebra. (A): 1 month; (B): 3 months; (C): 6 months.

Long-term DSA follow-up of different surgeries was shown in Figs 6–8. The morphologies and positions of PMMA cements in the fractured vertebra showed no obvious changes during the 6-month follow-up. In addition, the upper and lower endplates of the fractured vertebra also showed no obvious changes. Therefore, all the results demonstrated the three types of surgeries had stable height restoration ability.

## Discussion

Osteoporosis and associated fractures are common in clinics. Minimally invasive treatment has been increasingly used and will be the mainstream method for VCF treatment. Currently, the two main minimally invasive techniques are PVP and PKP. However, the benefits and shortcomings of these two techniques are still debated such as height restoration and bone cement leakage [12–14]. PMCP is an emerging minimally invasive technique for the treatment of VCFs and is developed by referring to PVP and PKP.

In this work, the clinical behaviors of PVP, PKP, and PMCP were accessed. PVP, PKP, and PMCP treatments had significant and similar ability in pain relief and functional recovery for the treatment of VCFs (Table 4). The differences among them lay in the bone cement leakage (Table 2), bone cement distribution (Table 2), and height restoration (Table 3).

It is well known that bone cement leakage may occur in PVP and PKP treatment. The bone cement leakage may lead to pulmonary embolism [15–17], neurological deficit [18], and even paraplegia [19]. This work showed PMMA leakage occurred in 6.67% patients treated by PVP, 3.33% patients treated by PKP, and none of patients treated by PMCP (Table 2). The difference might be resulted from the technical difference of these three treatments. Due to possible tissue disruption such as a cleft in the fractured vertebral body, the high bone cement perfusion pressure of PVP technique may induce the cortical, epidural, and anterior venous cement leakage [20]. The cavity created in PKP treatment may decrease the bone cement perfusion pressure compared with PVP treatment, which may decrease the possibility of bone cement leakage. However, PMMA cement leakage can occur if the surrounding bone tissue is broken such as the broken endplate (Fig 5). The mesh container in PMCP treatment keeps PMMA cement inside the container and only partial cement leaks outside from the mesh to bone trabeculae, and therefore, no PMMA cement leakage occurs in PMCP treatment even if the surrounding tissue is broken. Therefore, PMCP treatment had a better inhibition ability of bone cement leakage than both PVP and PKP treatments.

Depending on the shape of cement after surgeries, the cement patterns can be divided into three categories [21–23]: (i) solid patterns, in which cement forms a mass; (ii) trabecular patterns, in which cement spreads along the fine bone trabeculae and intersperseds in the trabeculae; and (iii) mixed patterns, in which cement forms a mass with a spreading along the fine bone trabeculae. In this work, the cement patterns after the PVP, PKP, or PMCP treatments were summarized in Table 2. Three (solid, trabecular, and mixed patterns), two (solid and mixed patterns), and one (mixed patterns) types of cement patterns were observed in PVP, PKP, and PMCP groups, respectively. The difference might be resulted from the technical difference of these three treatments. In PVP treatment, the puncture needle can be placed in two different areas in fractured vertebral body, which induces different patterns of cement [23]. When the puncture needle is placed in cleft areas in PVP treatment, PMMA cement tends to fill them without further diffusion, making a solid pattern. When the puncture needle is placed in trabeculae in PVP treatment, PMMA cement tends to fill with a trabecular pattern first and then extends into the cleft areas, making a mixed pattern. Therefore, three types of cement patterns (solid, trabecular, and mixed patterns) could be observed in PVP group (Table 2). In PKP treatment, although the puncture needle can be placed in two different areas in the fractured vertebral body, the inflation of commercial inflatable bone tamp or Sky-bone expander creates a cavity and pressures the surrounding bone tissues. If the surrounding bone tissues are completely compacted, PMMA cement tends to fill the cavity without further diffusion, making a solid pattern. However, if the surrounding bone tissues are not completely compacted, PMMA cement tends to fill the cavity and then diffuse into trabecular, making a mixed pattern. Therefore, two types of cement patterns (solid and mixed patterns) could be observed in PKP group (Table 2). In PMCP treatment, the cavity is created by expanding the spring leaves and rotating the bone expansion brace to cut the bone tissues. The surrounding bone tissues are not compacted. Therefore, PMMA cement can diffuse into trabecular after leaking outside of the mesh container, making a mixed pattern. Therefore, only mixed patterns of bone cement could be observed in PMCP group (Table 2). Previous work showed PVP and PKP may induce vertebra refracture after a long time such as one year, which is commonly associated with solid or trabecular cement pattern.[24, 25] Therefore, mixed cement pattern is preferred for the treatment of VCFs. Compared with PVP and PKP treatments, PMCP treatment had a controlled cement pattern. It might decrease the potential risks of vertebra refractures.

Height restoration of the treated fractured vertebral body is an important parameter to evaluate the clinical efficacy for minimally invasive techniques. Most previous studies indicated that PVP only could achieve partial restoration of the fractured vertebral body height [26]. Our previous work also showed PVP couldn’t improve the height of fractured vertebral body with interaosseous cleft [12]. This work also confirmed that the height restoration ability of PVP treatment was not ideal (Table 3). Therefore, PVP could not restore geometric and loading alignments of the fractured vertebral bodies, decrease their additional buckling torque, and recover the equilibrium dispersive ability of the intervertebral disc to bearing load [27, 28]. The incomplete restoration of the fractured vertebral body height increases the potential risk of vertebral refractures [2, 26, 29–31]. There is wide agreement that the height restoration ability of PKP is better than that of PVP due to the use of commercial inflatable bone tamp or Sky-bone expander. This work also confirmed this agreement (Table 3). Moreover, this work showed that the height restoration ability of PMCP was similar to PKP and better than PVP (Table 3), which might be resulted from the inflation of mesh container. Therefore, PVP, PKP, and PMCP treatments had obvious height restoration ability. Moreover, PKP and PMCP treatments had better height restoration ability.

This study suffers from several limitations. Most importantly, this study was retrospective in nature. Secondly, following up radiographs were not obtained, the change of post-procedure vertebral height and wedge angle could not be analyzed. Finally, only a small patient sample size was available for this study. However, to the best of our knowledge, this study represents the largest series of patients with VCFs treated with PVP, PKP and PMCP. A prospective study of a larger group of patients may show a higher complication rate, especially bone cement leakage.

To sum up, for the treatment of VCFs, PVP, PKP, and PMCP had significant and similar ability in pain relief and functional recovery. PMCP treatment had a better inhibition ability of bone cement leakage than both PVP and PKP treatments. Three (solid, trabecular, and mixed patterns), two (solid and mixed patterns), and one (mixed patterns) types of cement patterns were observed in PVP, PKP, and PMCP groups, respectively. In addition, PVP, PKP, and PMCP treatments had obvious height restoration ability. Moreover, PKP and PMCP treatments had better height restoration ability. These results indicate that minimally invasive techniques were effective methods for the treatment of VCFs. Moreover, these initial outcomes suggest PMCP treatment was better than both PVP treatment and PKP treatment. The surgical process of PMCP is similar to PKP and is simple, convenient, safe, and effective. Further work will focus on the long-term observation of the implanted mesh container in the treated vertebral body.

## Materials and Methods

The methods were carried out in accordance with the approved guidelines, which followed the Declaration of Helsinki. All the experimental protocols were approved by the Ethics Committee of the General Hospital of Jinan Command. The PMCP mesh container is made of polyethylene terephthalate and has a mesh size of 80 (Fig 3B).

### Patients Selection and Experimental Design

In order to avoid the mutual interference of multiple fractured vertebral body lesions, only patients with single VCF were selected. 90 patients (PVP:PKP:PMCP = 1:1:1) with single VCF were treated with PVP, PKP, and PMCP in a randomized double-blind way at the General Hospital of Jinan Command between June 2010 and March 2015. The patients were randomly assigned to these three groups by random numbers table [32]. Two independent blinded radiologists performed the surgeries of PVP, PKP, or PMCP. Three independent blinded radiologists assessed the radiographs, measured vertebral height and wedge angle, and finished the pain and function evaluation of patients. We performed a retrospective review of these patients treated with PVP (30 patients), PKP (30 patients), and PMCP (30 patients) for this study. All patients were given written informed consent before the procedures. The patients’ medical records and radiographic studies were acquired with approval from the institutional review board of the General Hospital of Jinan Command. The clinical characteristics of the 90 patients with single VCF were summarized in Table 1.

### Image assessment

Before the procedures, all patients were subjected to a physical examination of percussion pain over the spine in order to determine the symptomatic vertebral level. Dual energy X-ray absorptiometry was applied to evaluate the osteoporosis level. Medical imaging examinations such as X-ray plain film, CT, and/or MR were performed to evaluate the location and severity.PVP, PKP, PMCP techniques.

Sedation and analgesia were given just before the surgeries. The patient was placed in a prone position on the angiographic table. After confining the fractured vertebra body and its corresponding pedicles to be treated with fluoroscopy, local anaesthesia was treated by administering 1% lidocaine to the periosteum of the targeted pedicle. An 11- or 13-gauge bevelled puncture needle was advanced into the fractured vertebra through a unilateral transpedicular (in 16 levels) approach. Then PVP, PKP, or PMCP were performed for the patients. PVP (Fig 1) was performed by staff radiologists who used a modified form of the method described by Jensen et al.[33]. Briefly, PMMA bone cement was prepared by mixing powder component with liquid component until it formed into a paste with high viscosity. The cement was loaded into a bone cement perfusion apparatus and manually injected under fluoroscopic guidance. The injection was stopped when the cement reached the posterior quarter of the fractured vertebral body or had a potential tendancy of cortical, epidural, and anterior venous cement leakage. PKP (Fig 2) was performed by staff radiologists who used a modified form of the method described by Lieberman et al.[34]. Briefly, a bone drill was placed in to drill a circular hole in the fractured vertebral body as a working channel. After withdrawing the bone drill, an inflatable bone tamp (Shandong Guanlong Medicial utensils Co., Ltd., Jinan City, Shandong Province, China) was placed into the working channel and was slowly inflated by applying a force pump with a pressure gauge. The inflation was stopped when the maximum pressure or the maximum inflatable volume was reached. The inflatable bone tamp was withdrawn and a cavity was formed in the fractured vertebral body. PMMA cement was manually injected into the cavity by applying a bone cement perfusion apparatus under fluoroscopic guidance. The injection was stopped when the cement reached the posterior quarter of the fractured vertebral body or had a potential tendancy of cortical, epidural, and anterior venous cement leakage. PMCP was performed by staff radiologists who used a method described as follows (Fig 4). Briefly, a bone drill was placed in to drill a circular hole in the fractured vertebral body as a working channel. After withdrawing the bone drill, an expansion brace (Shandong Guanlong Medicial utensils Co., Ltd., Jinan City, Shandong Province, China) was placed into the working channel. The surrounding bone tissues were cut by expanding the spring leaves and rotating the expansion brace, and therefore, a cavity was formed in the fractured vertebral body. After withdrawing the expansion brace, a mesh container (Shandong Guanlong Medicial utensils Co., Ltd., Jinan City, Shandong Province, China) was advanced into the cavity and PMMA cement was manually injected into the mesh container by applying a bone cement perfusion apparatus under fluoroscopic guidance. With the continuous injection of PMMA, the mesh container was inflated and the height of the fractured vertebral was restored. At a certain injection amount, PMMA bone cement leaked outside the mesh container from the meshes and entered into bone trabeculae (Fig 4F). The injection was stopped when the cement reached the posterior quarter of the fractured vertebral body or had a potential tendancy of cortical, epidural, and anterior venous cement leakage. After surgeries, CT was performed immediately to assess PMMA cement leakage in each case.

### Measurements of vertebral height and wedge angle

The surgical procedures were performed with C-arm digitalized x-ray system (Angiostar, Siemens, Germany; Innova 4100, GE, USA) at a 40-cm field of view and automatic adaptation of kV, mA, and time of exposure. The fluoroscopic spot radiographs were exported to a workstation (Advantage Windows 4.0; GE, USA). The radiographs were reviewed to measure the anterior and middle vertebral heights before and after the surgeries. Then, the restoration percentage of the anterior and middle vertebral heights was calculated as follows: [35]

$$R_{p}=\frac{\left(H_{a}-H_{b}\right)}{{\text{H}}_{b}}\times 100\%$$ (1)

where R_p_ is the restoration percentage of the anterior or middle vertebral height after the surgery, H_a_ is the anterior or middle vertebral height after the surgery, H_b_ is the anterior or middle vertebral height before the surgery.

The wedge angles were measured as described in our previous report [12]. Briefly, the wedge angle was measured as the angle between the upper endplate and the lower endplate of the treated vertebral body in the standard lateral fluoroscopic spot radiographs before and after the surgeries.

### Pain and function evaluation

Pain level of patients before and 3 d, 1 m, 3 m, 6 m after the surgeries was assessed according to the visual analogue scale (VAS) score, a pain scale between 0 and 10, where 0 indicates no pain and 10 indicates the worst pain imaginable. Function level of patients before and 3 d, 1 m, 3 m, 6 m after the surgeries was assessed according to The Oswestry Disability Index (ODI).

### Statistical analysis

All data were presented as the mean value ± standard deviation (SD). Statistical comparisons were performed using a paired *t* test or a χ^2^ test, with a *P* value of less than 0.05 considered statistically significant. The SPSS software package was used for statistical analysis.
